# KAP1 phosphorylation promotes the survival of neural stem cells after ischemia/reperfusion by maintaining the stability of PCNA

**DOI:** 10.1186/s13287-022-02962-5

**Published:** 2022-07-07

**Authors:** Wan Wang, Tianqing Yan, Xinjian Guo, Heng Cai, Chang Liang, Linyan Huang, Yanling Wang, Ping Ma, Suhua Qi

**Affiliations:** 1 School of Medical Technology, Xuzhou Key Laboratory of Laboratory Diagnostics, Xuzhou Medical University, Xuzhou, 221004 China; 2 Pharmacology College, Xuzhou Medical University, Xuzhou, 221004 China; 3 School of Basic Medical Science, Xuzhou Medical University, Xuzhou, 221004 China; 4 Department of Laboratory Medicine, Affiliated Hospital of Xuzhou Medical University, Xuzhou, 221002 China

**Keywords:** Cerebral ischemia/reperfusion (I/R), Neural stem cells (NSCs), Proliferation, KRAB domain protein 1(KAP1), PCNA

## Abstract

**Aims:**

To explore the function of phosphorylation of KAP1 (p-KAP1) at the serine-824 site (S824) in the proliferation and apoptosis of endogenous neural stem cells (NSCs) after cerebral ischemic/reperfusion (I/R).

**Methods:**

The apoptosis and proliferation of C17.2 cells transfected with the p-KAP1-expression plasmids and the expression of proliferation cell nuclear antigen (PCNA) and p-KAP1 were detected by immunofluorescence and Western blotting after the Oxygen Glucose deprivation/reperfusion model (OGD/R). The interaction of p-KAP1 and CUL4A with PCNA was analyzed by immunoprecipitation. In the rats MCAO model, we performed the adeno-associated virus (AAV) 2/9 gene delivery of p-KAP1 mutants to verify the proliferation of endogenous NSCs and the colocalization of PCNA and CUL4A by immunofluorescence.

**Results:**

The level of p-KAP1 was significantly down-regulated in the stroke model in vivo and in vitro. Simulated p-KAP1(S824) significantly increased the proliferation of C17.2 cells and the expression of PCNA after OGD/R. Simulated p-KAP1(S824) enhanced the binding of p-KAP1 and PCNA and decreased the interaction between PCNA and CUL4A in C17.2 cells subjected to OGD/R. The AAV2/9-mediated p-KAP1(S824) increased endogenous NSCs proliferation, PCNA expression, p-KAP1 binding to PCNA, and improved neurological function in the rat MCAO model.

**Conclusions:**

Our findings confirmed that simulated p-KAP1(S824) improved the survival and proliferation of endogenous NSCs. The underlying mechanism is that highly expressed p-KAP1(S824) promotes binding to PCNA, and inhibits the binding of CUL4A to PCNA. This reduced CUL4A-mediated ubiquitination degradation to increase the stability of PCNA and promote the survival and proliferation of NSCs.

**Supplementary Information:**

The online version contains supplementary material available at 10.1186/s13287-022-02962-5.

## Introduction

Stroke is a significant cause of mortality and disability worldwide [1]. Ischemic stroke accounts for more than 87% of stroke events and is an acute cerebral arterial obstruction characterized by abrupt onset and focal neurological deficits [2]. Cerebral ischemia–reperfusion (I/R) injury after vascular recanalization further amplifies the death of nerve cells [3]. Therefore, strategies to promote recovery from stroke require enhanced neurogenesis to compensate for damaged nerve cells. Numerous studies have explored the use of neural stem cells (NSCs) for stroke treatment, as NSCs can proliferate and differentiate into neurons and glial cells to reconstruct destroyed neuronal circuits [4–8]. With the continued development of stem cell technology, the transplantation of exogenous NSCs has been used to treat various central nervous system diseases [9–11]. However, exogenous NSCs therapy remains limited such as transplant rejection, poor stability, difficulty in getting materials, and ethical controversies [12, 13]. Given these factors, it is essential to investigate how to enhance the proliferation of endogenous NSCs to improve survival and recovery from neurological deficits after cerebral I/R.

**Fig. 1:**
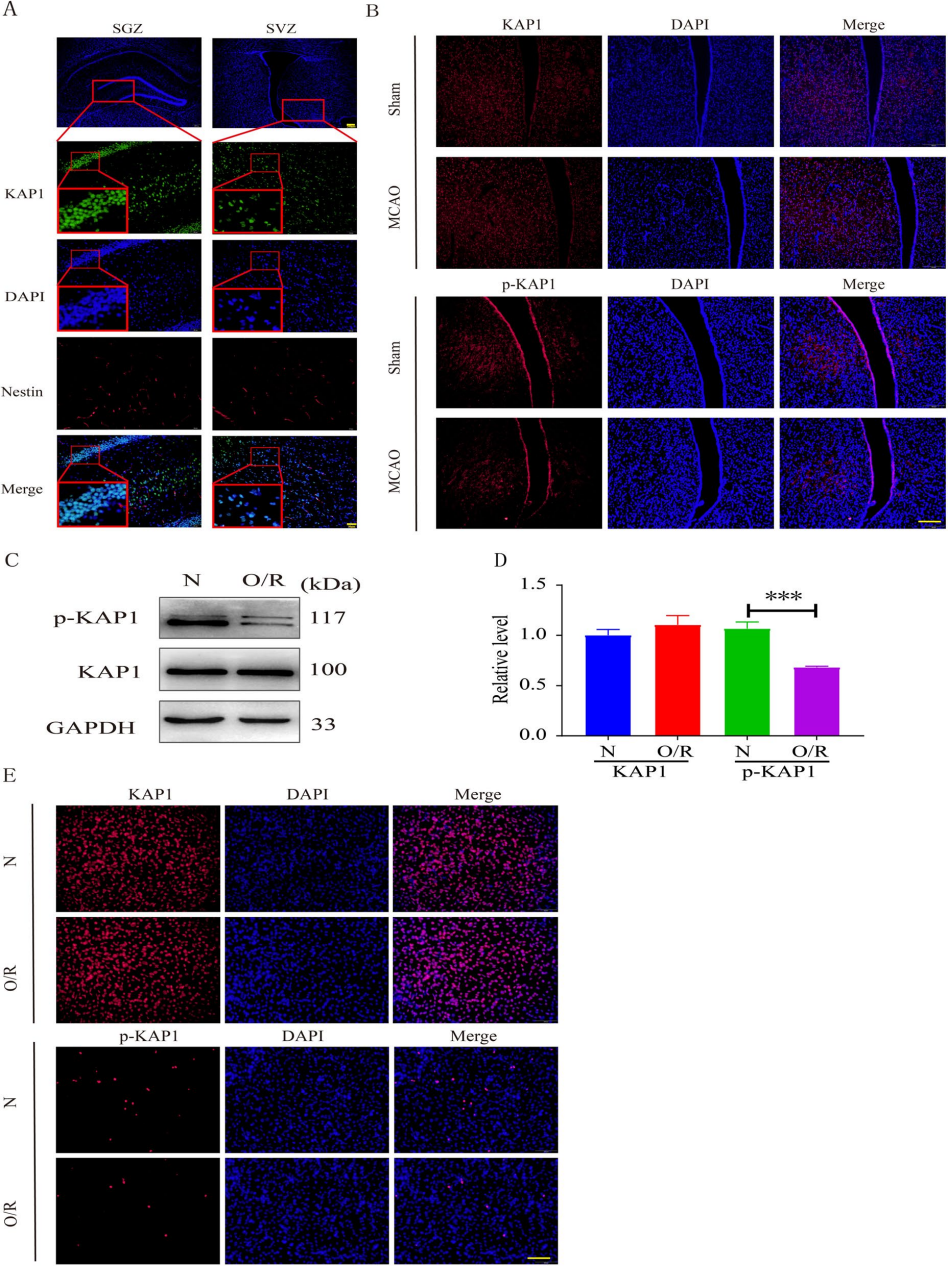
The expression of KAP1 and p-KAP1 in NSCs after cerebral I/R. **A** The expression and location of KAP1 in the subgranular zone (SGZ) and Subventricular zone (SVZ) were examined by immunofluorescence staining. KAP1 was dyed red, Nestin was green and the nuclear was stained blue with DAPI. 100× , scale bar = 200 μm; 200 × , scale bar = 50 μm. **B** The phosphorylation and protein expression of KAP1 in SVZ of rat subjected to I/R were analyzed by immunofluorescence staining. 200 × , scale bar = 200 μm. **C** Western blot analysis was also utilized to detect the phosphorylation and protein expression levels of KAP1 in C17.2-NSCs after OGD/R. **D** Bands were scanned, and the intensities were represented as folds of normoxia group, ^***^P < 0.001 versus normoxia group (N means normoxia, O/R means OGD/R). Data were expressed as mean ± SEM (*n* = 5). **E** Immunofluorescence staining was also performed to detect phosphorylation and protein expression. 200 × , scale bar = 100 μm

The Krueppel-associated box (KRAB) is a domain of around 75 amino acids that are found in the N-terminal part of about one-third of eukaryotic Krueppel-type C2H2 zinc finger proteins (ZFPs) [14]. KRAB domain protein 1(KAP1), also known as TRIM28 (tripartite motif-containing protein 28) or TIF1β (transcriptional intermediary factor 1β), is mainly localized in the cellular nucleus (Fig. 1A) and is a transcriptional cofactor that was initially isolated, cloned, and purified by affinity chromatography in 1996 [15]. KAP1 is involved in regulating of the embryonic stem cell cycle and control of proliferation and apoptosis [16, 17]. Post-translational modifications of KAP1, including phosphorylation and ubiquitination, are critical for its function in stem gene expression, embryonic development, immune regulation, tumor occurrence and development, and DNA damage response [18, 19]. The phosphorylation of KAP1 (p-KAP1) acts as an interpreter of cell signaling and modulates the ability of MyoD to drive myogenesis [20]. The p-KAP1 occurred at Tyr-449, Tyr-458, and Tyr-517 residues inhibit the association of KAP1 and heterochromatin protein 1α with heterochromatin [21]. Additionally, the p-KAP1(S824) has been reported to participate in embryonic stem cell proliferation and differentiation, maintaining stemness gene expression [22]. However, whether KAP1 and its phosphorylation state can regulate the functions of NSCs has not been reported.

Proliferating cell nuclear antigen (PCNA), a marker of the proliferation of NSCs, is a critical factor in DNA replication and cell cycle regulation [23]. Increased expression of PCNA indicates increased proliferation ability of NSCs, required for successful clinical treatment of central nervous system diseases [24, 25]. Jang et al. reported that p-KAP1(S473) could interact with PCNA to restore heterochromatin after DNA replication during the S phase [26]. Additionally, p-KAP1 recruits DNA repair-related factors after damage to facilitate DNA repair [27]. Therefore, we hypothesized that there might be a relationship between the phosphorylation level of KAP1 and the proliferation of NSCs after cerebral I/R injury so that strategies targeting p-KAP1 could be used to prevent or treat ischemic stroke.

In this study, we found that p-KAP1 at serine 824 (S824) could promote the proliferation of NSCs and reduce apoptosis and neurobehavioral deficits, thereby promoting the recovery of cerebral I/R-induced neurological dysfunction. The underlying mechanism is that p-KAP1(S824) binds to PCNA and inhibits the binding of PCNA to E3 ubiquitin ligase CUL4A, reducing CUL4A's degradation of PCNA and thereby promoting the survival and proliferation of NSCs during I/R. Analysis of the KAP1/PCNA signaling pathway suggested that p-KAP1 may be a target for endogenous neurogenesis in cerebral I/R treatment.

## Materials and methods

### Culture of the C17.2 cells and establishment model of OGD/R

The C17.2-NSCs was purchased from the American Type Culture Collection (Rockville, MD, USA) and were cultured in Dulbecco's modified Eagle's medium (DMEM; Gibco, Carlsbad, USA) with 10% (v/v) fetal bovine serum (FBS; Gibco, Carlsbad, USA) in a humidified incubator with 5% CO_2_ and 95% air at 37 °C. In the Oxygen Glucose deprivation/reperfusion model (OGD/R), cells were washed three times with sterilized PBS, and the medium was replaced with a sugar-free medium culturing in O_2_/CO_2_ (1% / 5%) three-gas incubator. After six hours, the cells medium was replaced with DMEM containing 10% FBS for another 24 h in a humidified incubator with 5% CO_2_ and 95% air at 37 °C.

### Adeno-associated virus-mediated KAP1 mutations and plasmid transfection

Adeno-associated virus-mediated KAP1 (AAV2/9-KAP1-GFP) and its mutations (AAV2/9-KAP1-S824A-GFP, AAV2/9-KAP1-S824D-GFP) were purchased from Shanghai Sangon Biological Engineering Technology & Services Co., Ltd. 2μL of AAV-mediated KAP1 and its mutations (injected at a titer of 10^9^ VG/μl) were administered to the rats intracerebroventricularly (i.c.v) (bregma, 1.5 mm lateral, 0.8 mm posterior, and 3.8 mm deep) 14 days before ischemia. A total of 50 rats were stochastic fall into 5 groups: the (1) NC (Scramble AAV 2/9), (2) NC + I/R (Scramble AAV 2/9 + I/R), (3) WT + I/R (AAV2/9-KAP1 + I/R), (4) S824D(AAV2/9-KAP1-S824D + I/R), (5) S824A(AAV2/9-KAP1-S824A + I/R) groups (Fig. 6A). KAP1 plasmids (WT, S824D, S824A) were obtained from Shanghai Sangon and were transiently transfected into cells with Lipo3000 liposome transfection reagent following the manufacture’s protocols. Briefly, 60–80% confluent cells were prepared for transfection. Abandon the medium and wash with PBS 3 times. Then, add Opti-MEM into every well. Next, prepare lipofectamine 3000 reagents and plasmid mixture. The corresponding plasmid DNA (including P3000) and liposome were diluted with Opti-MEM medium, respectively, according to the size of the perforated plate. Then the two were mixed after standing at room temperature for 5 min. After that incubate at room temperature for 15 min. Finally, the plasmid DNA-liposome complex was added to the cells in each well and cultured in the cell incubator for 24–48 h. The transfection efficiency was confirmed by Western blot analysis.

### Western blotting

Cells were lysed in RIPA lysis buffer (Beyotime Biotechnology, Shanghai, China) containing 1% protease inhibitor cocktail (VICMED, VPI012), and the cell lysates were then centrifuged at 14,000 × g at 4 °C for 15 min. Protein concentrations were quantified using the Bicinchoninic Acid protein assay kit (BCA; Beyotime, P0010). Total proteins (50 μg) were separated by SDS-PAGE and electro-transferred to polyvinylidene difluoride membranes (PVDF; Millipore, Billerica, MA, USA). Then the membranes were blocked with TBST buffer (NaCl 150 mmol/L, Tris 10 mmol/L, Tween-20 0.05% (v/v) pH = 7.6) containing 5% nonfat milk powder at room temperature for 2 h. The membranes were then probed overnight at 4 °C with primary antibodies, including phospho-KAP1(1:1000, Abcam, ab70369), KAP1(1:1000, Cell Signaling Technology, #4123), PCNA (1:2000, Servicebio, GB11010), Bcl-2 (1:1000, ABclonal, A0208), Bax(1:1000, ABclonal, A0207) followed by incubation with corresponding HRP-conjugated secondary antibodies (1:5000, Proteintech, SA00001-2) at room temperature for 2 h. Proteins on the membranes were visualized by adding the enhanced chemiluminescence reagent (Millipore, USA). Band intensities were analyzed using Image J 1.25 software (National Institutes of Health, Bethesda, USA) and normalized to loading controls.

### Immunoprecipitation

Immunoprecipitation experiments were carried out with protein lysates using Beyotime Western and IP cell lysis buffer containing a 1% protease inhibitor cocktail (VICMED, VPI012). The cell lysates were then centrifuged at 14,000 × g at 4 °C for 15 min. Protein concentrations were quantified using the Bicinchoninic Acid protein assay kit (BCA; Beyotime, P0010). Add a certain amount of the antibody into the protein lysates and shake at 4 ℃ overnight. The next day, wash the protein A/G magnetic beads (MCE) with 0.5% PBST (1 × PBS + 0.5% Triton X-100) three times and then add it into the protein antigen–antibody mixture. Then, continue to shake at 4 ℃ for 2.5 h. After that wash the protein A/G magnetic beads-protein antigen–antibody complex with 0.5% PBST five times. Finally, add 20–30 μl 2 × protein loading buffer into the complex, and then heat it in 100 ℃ water for 5 min. Samples were centrifuged at 12,000 g for 2 min, and the supernatant was collected. Primary antibodies used in this stage including Flag (1:100, Proteintech, 20543-1-AP), KAP1 (1:100, Cell Signaling Technology, #4123), PCNA (1:100, Abcam, ab29), PCNA (1:2000, Servicebio, GB11010), Flag (1:10,000, D110005, Sangon Biotech), CUL4A (1:500, Proteintech, 66038-1-Ig), Ubiquitin (1:1000, Cell Signaling Technology, #3936T), Ubiquitin (linkage-specific K63, 1:1000, Abcam, ab179434), Ubiquitin (linkage-specific K48, 1:1000, Abcam, ab140601), α-Tubulin (1:1000, Beyotime Biotechnology, AF0001).

### CCK-8 cell proliferation assay

The cells transfected with mutant plasmids were grown in a 96-well plate. After attachment, cells were subjected to OGD/R or not. Cell Counting Kit-8 reagent (CCK-8, Beyotime, Jiangsu, PR China) and DMEM medium were mixed in 1:10. 100 μl of the mixture was added to each well and then incubated in a 5% CO_2_ incubator at 37 ℃ for 0.5–1 h. Then, the absorbance was determined at 450 nm with a microplate analyzer.

### Bromodeoxyuridine (BrdU) staining

The cells transfected with mutant plasmids were grown in a 24-well plate. After attachment, cells were subjected to OGD/R or not and were treated with or without BrdU for eight hours before staining. BrdU staining steps were as follows. Firstly, cells were washed with 0.3% TBS for 5 min × three times. Then, fix with 4% paraformaldehyde for about 20 min at room temperature. Next, cells were washed with TBS for 5 min × three times. Subsequently, 2 N hydrochloric acid was added to cells denaturing at room temperature for 1 h. After that wash with TBS for 5 min × three times, and seal 1 h at room temperature (sealing fluid is 10% goat serum + 0.3% TritonX-100 PBS). Finally, incubate cells with the anti-BrdU antibody (1:500, Abcam, ab152095) at 4 ℃ overnight (diluent with 1% goat serum + PBS + 0.3% TritonX-100). The next day, wash with TBS for 5 min × three times and incubate with secondary antibody (1:1000, Invitrogen, A-21207) at room temperature for two hours. Then, wash with TBS for 5 min × 3 times and stain with DAPI dye for 10 min. Finally, wash with TBS for 5 min × 3 times and photograph under Olympus IX73 inverted fluorescence microscope.

### Annexin V-APC/7-AAD apoptosis assay

The cells transfected with mutant plasmids were grown in middle-plates, followed by OGD/R treatment or not after adherence. Digest and collect with trypsin free of EDTA, and wash with PBS twice to collect 1–5 × 10^5^ cells. Cells were suspended with 500 μl binding buffer. Five μl Annexin V-APC and 5 μl 7-Aminoactinomycin D (7-AAD) dye were added to cells. Incubate at room temperature in the dark for 5–15 min. Then, samples were detected with Flow cytometry within one hour.

### Quantitative real-time fluorescent polymerase chain reaction (qRT-PCR)

The procedures of real-time PCR were the same as in our previous work [28]. Primer sequences employed in this study are listed: PCNA primers: sense, TTTCACAAAAGCCACTCCACTG; antisense, CTTTAAGTGTCCCATGTCAGCAAT. GAPDH primers (used as internal control): sense, TGTTCGTCATGGGTGTGAACC; antisense, GCAGTGATGGCATGGACTGTG.

### Establishment of rat middle cerebral artery occlusion (MCAO) model

Male Sprague–Dawley rats (Beijing Vital River Laboratory Animal Technology Co., Ltd.) aged between 7 and 8 weeks old (180–200 g) were used in this study. Rats were housed in the vivarium managed by the Division of Animal Lab Center of Xuzhou Medical University with a 12 h light/dark cycle and ad libitum access to food and water.

The MCAO model refers to the established method [29] in the laboratory, and the specific steps are as follows. After isoflurane inhalation anesthesia, the rat’s neck was vertically cut with scissors. Separate the surrounding tissue and expose the rat’s left common carotid artery (CCA), external carotid artery (ECA), and internal carotid artery (ICA). Then, fasten CCA and ECA near the heart with a sutures clip and ICA with an  arteriole clip. Subsequently, a small incision was made with ophthalmic scissors at an oblique 45° between the CCA ligation point and the three fork-lets at the upper end. The standard cord plug corresponding to body weight was carefully inserted at the incision point. The micro-arteriole was loosened, the direction of the inlet line was adjusted to avoid the pterygopalatine artery, and the ICA was entered until the beginning of the middle cerebral artery (MCA). After ischemia for 1.5 h, the rats were under reperfusion for 24 h by pulling out the plug. The insertion depth of the threaded plug in the sham operation group was about 5 mm, and the rest of the treatment was the same as that in the model group.

### Balance beam test

The balance beam test was performed to assess the ability of rats to maintain balance and motor function while walking along an elevated and narrow strip of wood (122 × 2.5 × 42 cm). The performance was scored from 0 to 6 (no attempt to stay on the beam as 0 point); attempted to stay on the beam but no movement as 1 point; attempted to cross the beam but failed as 2 points; crossed the beam with contralateral hindlimb slips > 50% of the time as 3 points; crossed the beam with contralateral hindlimb slips < 50% of the time as 5 points; crossed the beam without slips as 6 points [30].

### Open field test

A square cage (1 m × 1 m, with walls 45 cm high) containing an opaque acrylic container and a video camera were applied in the test. A rat was placed in the center of the square cage. The locomotive activities, including travel distance, speed, and time in the corner, were recorded [31]. The whole arena was wiped clean with 75% ethyl alcohol between tests.

### Frozen section

Frozen sections of brain tissue were performed using a Leica freeze slicer. Firstly, the brain was removed from the sucrose solution, and the bottom of the brain was cut flat with a knife. Then, the back of the brain was placed facing the notch on the freezing platform and the abdomen facing the operator vertically. Wrap the cerebra from top to bottom with the embedding agent and freeze it in the machine for at least 2 h. After that put the cerebra in the freezer and cut it into 20 μm thick slices. The brain slices were then placed in phosphate buffer saline (PBS; NaCl 137 mmol/L, KCl 2.7 mmol/L, Na_2_HPO_4_·12H_2_O 8 mmol/L, NaH_2_PO_4_·2H_2_O 1.5 mmol/L). If stored for a long time, the slices needed to be placed in a PBS solution containing 60% glycerin and stored in a refrigerator at – 80 ℃.

### Immunofluorescence staining of frozen sections

The rats were sacrificed 7 days after the MCAO model, and the brain tissue was taken for a follow-up study. The rat brain slices attached to the viscous slide were washed with TBS (0.3% Triton X-100 PBS) for 5 min × two times and sealed with blocking solution (0.3% Triton X-100 + 10% PBS of goat serum) at room temperature for 1 h. The rat brain frozen sections were then added with primary antibody PCNA(1:100, Abcam, ab29), Nestin(1:100, Abcam, ab6142), Ki-67(1:100, Cell Signaling Technology, #9129), CUL4A(1:100, Proteintech, 66038-1-Ig), KAP1(1:100, Abcam, ab70369), phospho-KAP1(1:100, Abcam, ab70369), and incubated at 4 °C in a wet box overnight. The following day, wash the brain slides with TBS for 5 min × five times and incubate with goat anti-mouse IgG (H + L)(1:1000, Cell Signaling Technology, #4410) or goat anti-rabbit IgG (H + L)(1:1000, Abclonal, AS007)secondary antibody in a wet box at room temperature for 1–2 h. Afterward, wash the brain slides with TBS for 5 min × five times, and then DAPI staining was added to the brain slides, incubating for 10 min. Finally, wash the brain slides with TBS for 5 min × five times, seal the slide with a sealing tablet, and photograph under Olympus IX73 inverted fluorescence microscope.

### Statistical analysis

The statistical analysis of data was conducted using GraphPad Prism 7. Experimental data were expressed as Means ± SEM. Student’s T-test and One-way ANOVA were used for statistical analysis. Dunnet T-test was used for comparison between multiple experimental groups and a control group. The data were checked for normally distributed by the Kolmogorov–Smirnov test (with Dallal-Wilkinson-Lillie for P-value) before using parametric tests. *P* < 0.05 indicated that the difference was statistically significant.

## Results

### The total expression of KAP1 remained unchanged, but the level of p-KAP1 decreased after cerebral I/R

To explore the role of KAP1 in cerebral I/R injury, we established the rat MCAO model for immunofluorescence staining. Compared with the sham group, there was no noticeable difference in the overall expression of KAP1 in the MCAO group, but the expression level of p-KAP1 was significantly reduced (Fig. 1B, Additional file 1: Fig. S1A). Next, the OGD/R model of C17.2 cells was used to simulate cerebral I/R injury in vitro, and we again detected the expression of KAP1 and p-KAP1 by western blot. As shown in Fig. 1C and D, there was no difference in KAP1 expression in C17.2 cells between the normoxia group and the OGD/R group. However, compared with the normoxia group, the expression of p-KAP1 was significantly decreased in the OGD/R group, and this result was confirmed by immunofluorescence staining (Fig. 1E and Additional file 1: Fig. S1B). These results showed that the total expression of KAP1 remained unchanged, but p-KAP1 declined significantly during cerebral I/R.

### Simulated p-KAP1 improved the proliferation of endogenous NSCs after cerebral I/R

**Fig. 2:**
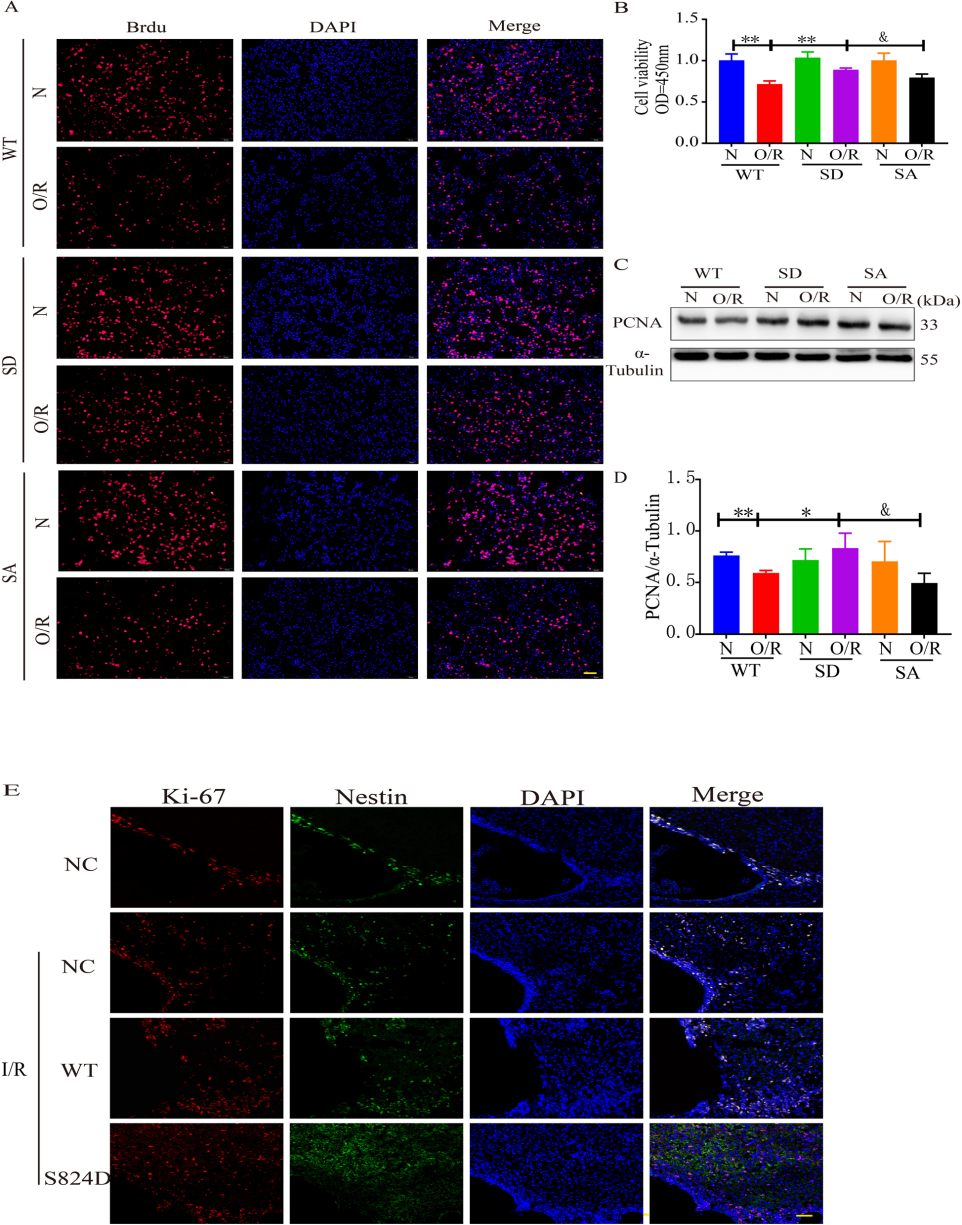
Effects of p-KAP1 on the proliferation of NSCs subjected to cerebral I/R. **A** BrdU immunofluorescence staining was performed to evaluate the C17.2-NSCs proliferation. 200 × , scale bar = 100 μm. **B** CCK-8 assay was also used to detect the proliferation of C17.2-NSCs. Data were presented as mean ± SEM (*n* = 3). ^**^P < 0.01 versus WT and O/R group; ^&^*P* < 0.05 versus SD and O/R group. **C**, **D** Western blot analysis was used to examine the expression of PCNA. Band intensities were represented as fold-values relative to the levels in normoxia treatment. Data were presented as mean ± SEM (*n* = 4), ^*^P < 0.05, ^**^P < 0.01 versus WT and O/R group; ^&^*P* < 0.05 versus SD and O/R group. **E** Ki-67 and Nestin colocalization immunofluorescence staining was performed to evaluate the neurogenesis of endogenous NSCs. 200× , scale bar = 100 μm

We next explored the function of p-KAP1 in endogenous NSCs. As shown in Additional file 1: Fig. S2, the proliferation ability of C17.2 cells was restored after 6 h of glucose and oxygen deprivation and reperfusion for 3 h, 6 h, or 12 h. But at 24 h of reperfusion, p-KAP1 and γ-H2AX (indicating DNA damage) were significantly down-regulated, and the expression of PCNA decreased at this time (Additional file 1: Fig. S2). We speculated that the reperfusion caused further cell damage at 24 h of reperfusion (i.e., reperfusion injury). The injury cannot be repaired by self-feedback. So we chose 24 h of reperfusion. We simulated phosphorylation of KAP1 (p-KAP1 is phosphorylated at serine 824. S824D and S824A, respectively, simulated the state of phosphorylation and non-phosphorylation) in vitro and in vivo. Using BrdU staining, we found that the proliferation ability of C17.2 cells transfected with wild-type (WT) KAP1 and KAP1 (S824A) in the OGD/R group was significantly lower than that of the normoxia group. Transfected with p-KAP1 (S824D) mimic, the decrease in C17.2 cell proliferation was reversed (Fig. 2A and Additional file 1: Figure S3). Cell viability in NSCs was measured by a CCK-8 kit. As shown in Fig. 2B and Additional file 1: Figure S4, the cell viability of transfected with p-KAP1 (S824D) mimic was significantly increased compared with that of KAP1(WT) or KAP1 unphosphorylated(S824A) after OGD/R. These results suggested that p-KAP1 (S824D) could improve cell viability after OGD/R. We also detected the expression of PCNA by Western blotting. OGD/R treatment led to down-regulation of PCNA expression in C17.2 cells, but the use of the p-KAP1 (S824D) mimic abrogated this down-regulation (Fig. 2C, D). We further analyzed the proliferation of endogenous NSCs in rat cerebra using immunofluorescence staining. The rats were transfected with adeno-associated virus-mediated KAP1 (AAV2/9-KAP1-GFP) and its mutations (AAV2/9-KAP1-S824A-GFP, AAV2/9-KAP1-S824D-GFP) 14 days before MCAO. As shown in Fig. 2E, compared with other groups subject to cerebral I/R, colocalization of Ki67 and Nestin in the Subependymal Ventricular Zone (SVZ) region was significantly increased in the viral-mediated KAP1 mutation (S824D) group. These results suggested that simulated p-KAP1 (S824) promoted the proliferation and regeneration of endogenous NSCs after cerebral I/R.

### Simulated p-KAP1 alleviated OGD/R-induced C17.2 cells apoptosis

**Fig. 3:**
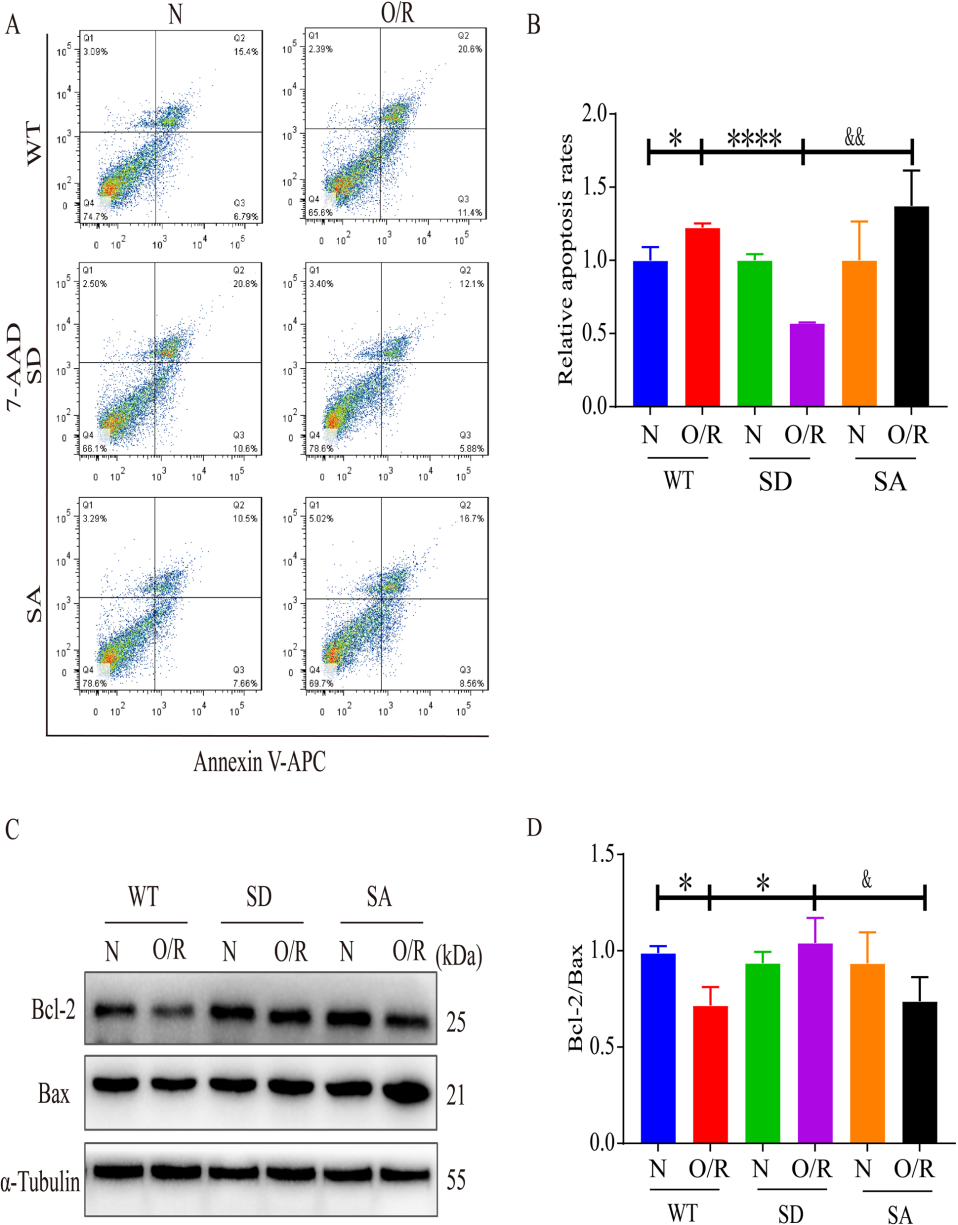
Effects of p-KAP1 on the apoptosis of C17.2-NSCs after OGD/R. **A**, **B** Annexin V-APC/7-AAD apoptosis detection kit was utilized to detect C17.2-NSCs apoptosis and the data were quantified as mean ± SEM (*n* = 3), ^*^P < 0.05, ^****^P < 0.0001 versus WT and O/R group; ^&&^*P* < 0.01 versus SD and O/R group. **C**, **D** Western blot analysis was used to examine the expression of Bcl-2/Bax and band intensities are represented as fold-values relative to the levels in normoxia treatment. Results were presented as mean ± SEM (*n* = 4), ^*^P < 0.05 versus WT and O/R group; ^&^*P* < 0.05 versus SD and O/R group

We then determined the relationship between p-KAP1 and apoptosis of C17.2 cells after OGD/R by Western blotting and flow cytometry. Flow cytometry results indicated that the apoptosis rates of C17.2 cells transfected with KAP1 WT or KAP1 S824A in OGD/R groups were higher than that of the normoxia group. In contrast, the KAP1 S824D group could reverse the apoptosis of C17.2 cells induced by OGD/R (Fig. 3A, B). We explored the possible pathways involved in the apoptosis of NSCs treated with p-KAP1 (S824) mimic. Western blotting showed the Bcl-2/Bax ratio of C17.2 cells was significantly decreased after exposure to OGD/R while simulating p-KAP1 could increase the ratio (Fig. 3C and D). These results indicated that the p-KAP1 mimic might reduce OGD/R-induced apoptosis of C17.2 cells through the Bcl-2/Bax signaling pathway.

### Simulated p-KAP1 enhanced binding to PCNA and decreased the interaction of CUL4A under cerebral I/R

**Fig. 4:**
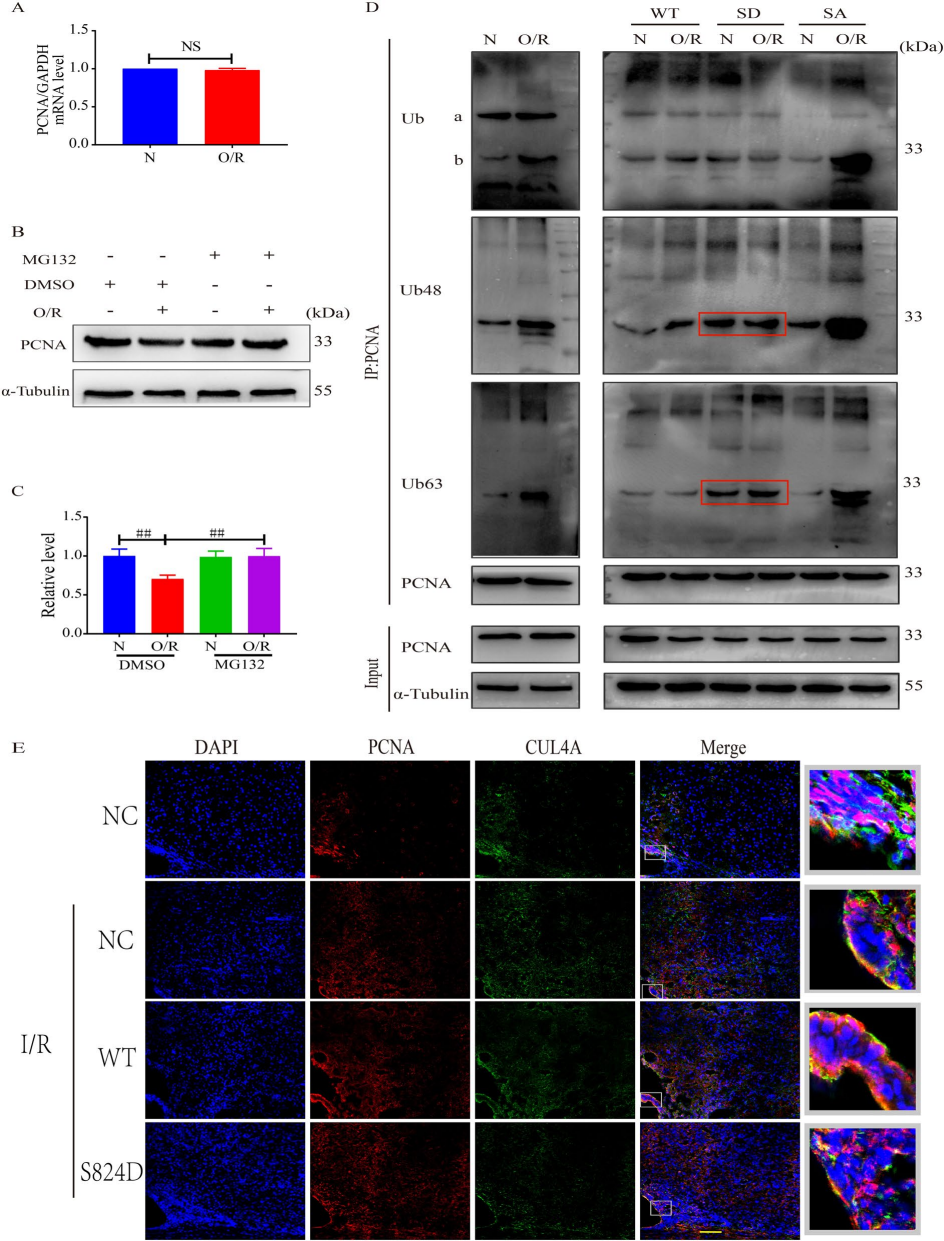
Effects of p-KAP1 on the stability of PCNA in NSCs undergoing cerebral I/R. **A** Real-time PCR analysis of PCNA mRNA levels in C17.2-NSCs after OGD/R. Data are the mean values ± SEM of three replicate experiments, NS indicates no significance. **B** Western blot analysis of PCNA protein levels in C17.2-NSCs after OGD/R. C17.2-NSCs were pre-incubated with 20 μM MG132 for 6 h before harvesting cells for protein lysis. **C** Band intensities are represented as fold-values relative to the levels in the normoxia group. Data were expressed as mean ± SEM (*n* = 4). ^##^*P* < 0.01 versus DMSO and O/R group. **D** C17.2-NSCs were treated with 20 μM MG132 for 6 h before harvesting for protein lysis and PCNA was precipitated with the anti-PCNA antibody. The protein levels and ubiquitination levels of PCNA in the immune complex were determined by Western blot. The protein levels of PCNA and *α*-Tubulin in cell lysates (input) were also analyzed by Western blot, and three independent experiments were performed. “a” indicates the heavy chain of IgG, “b” indicates the light chain of IgG. **E** PCNA and CUL4A colocalization immunofluorescence staining was performed to evaluate the neurogenesis of endogenous NSCs. 200× , scale bar = 100 μm

KAP-1 can act as an ubiquitin-like ligase of PCNA and mediate its ubiquitination degradation [32]. So, we assayed PCNA ubiquitination in endogenous NSCs after cerebral I/R. To do this, RT-PCR was used to detect the PCNA mRNA level. We found that both the normoxia group and the OGD/R group did not exhibit altered mRNA levels (Fig. 4A). C17.2 cells were treated with or without proteasome inhibitor MG132 (20 μM) for six hours, and then the expression of PCNA was detected. The down-regulation of PCNA in C17.2 cells induced by OGD/R was improved when pre-incubated with MG132 (Fig. 4B, C). We also performed immunoprecipitation and found that after OGD/R, the level of PCNA poly-ubiquitination at K48 and K63 sites increased in C17.2 cells, while the p-KAP1 mimic could alleviate this poly-ubiquitination (K48) (Fig. 4D). However, simulated p-KAP1(S824D) did not affect poly-ubiquitination (K63). This result indicates that the effects of p-KAP1 might depend on regulating the stability of PCNA by post-transcriptional modification, reducing its ubiquitination degradation by some ubiquitin enzymes. The expression of PCNA in the SVZ region of cerebral I/R rats was detected by immunofluorescence (Fig. 4E). As shown in Fig. 4E, the KAP1 mutation (S824D) mimic promoted its colocalization with PCNA and decreased the colocalization of PCNA with CUL4A in the SVZ region. The results in vivo were consistent with cellular experiments. Thus, we concluded that the p-KAP1 mimic might help regulate the proliferation of endogenous NSCs by influencing the stability of PCNA after cerebral I/R.

### Simulated p-KAP1 promoted the stability of PCNA and ultimately competitively inhibited the degradation of CLU4A to PCNA

**Fig. 5:**
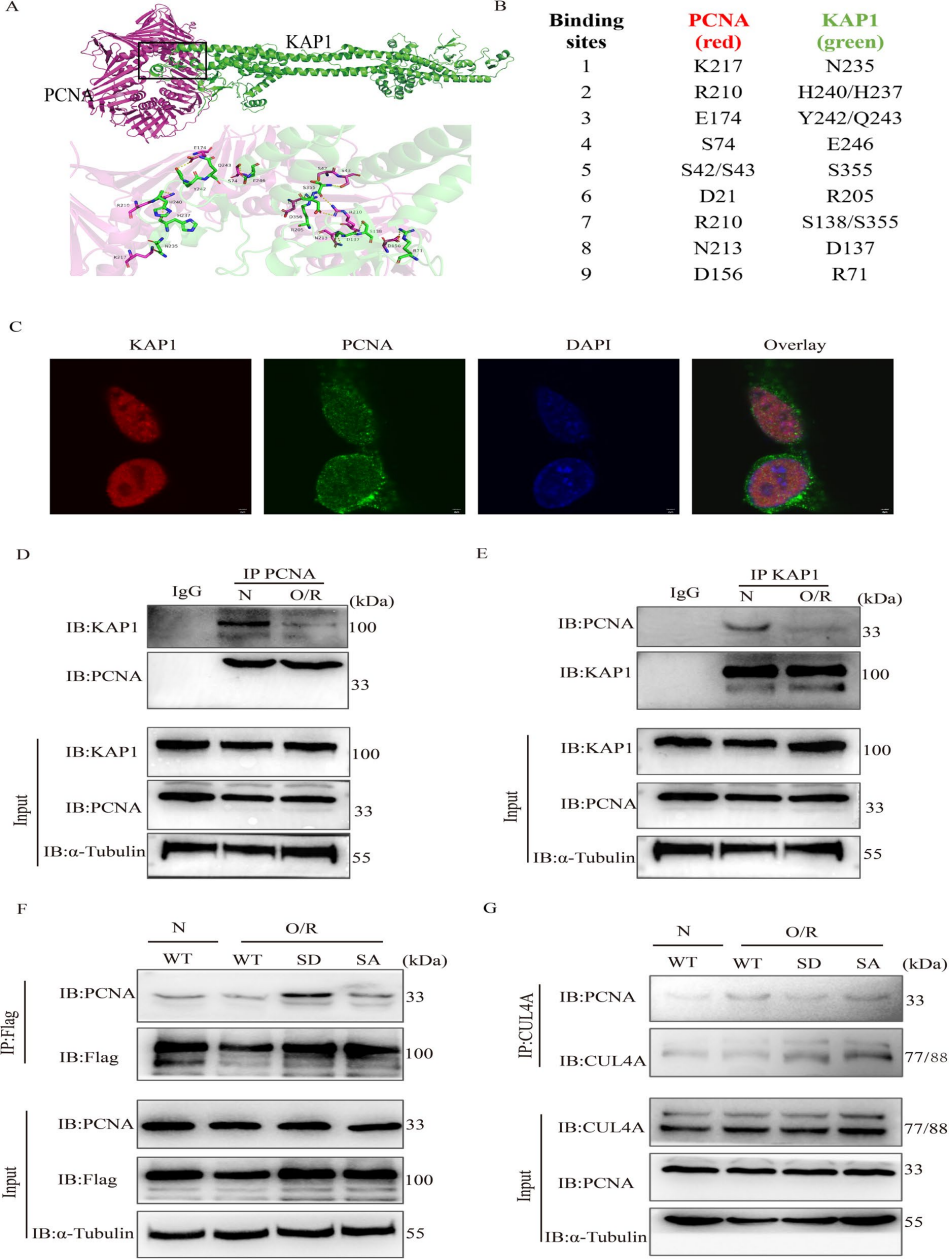
Effects of p-KAP1 on the binding of KAP1 to PCNA, PCNA to CUL4A in C17.2-NSCs after OGD/R. **A** Protein docking of KAP1(Green) and PCNA(Red). **B** Potential binding sites between KAP1 and PCNA. **C** Confocal immunofluorescence staining revealed the coexistence of KAP1 and PCNA in the cellular nucleus. Scale bar = 2 μm. **D**, **E** Immunoprecipitation and immunoblotting (IP–IB) were performed to confirm the association of KAP1 with PCNA in C17.2-NSCs subjected to 6 h of oxygen and glucose deprivation followed by 24 h of reoxygenation. **F** Site-mutant plasmids were transiently transinfected into C17.2-NSCs subjected to OGD/R, or no treatment, and the binding of KAP1 to PCNA was examined by IP–IB. **G** The binding of CUL4A to PCNA was investigated by IP–IB. The relative bands were selected from three independent experiments

To determine the regulatory relationship between p-KAP1 and PCNA in cerebral I/R, we studied their interaction by molecular docking and observed colocalization (Fig. 5A, B). Confocal microscopy showed that KAP1 colocalized with PCNA in the cellular nucleus (Fig. 5C). We also confirmed the interaction of KAP1 with PCNA in C17.2 cells by immunoprecipitation and found that this interaction decreased after OGD/R (Fig. 5D, E). For this condition, we found that simulated p-KAP1 could increase its association with PCNA (Fig. 5F). The binding of PCNA to its E3 ubiquitin ligase CUL4A increased in C17.2 cells exposed to OGD/R, while the p-KAP1 mimicked suppressed binding of CULA4 (Fig. 5G). In detail, the increased binding of PCNA to p-KAP1 resulted in decreased interaction of CUL4A, which weakened the ability of CUL4A to degrade PCNA, thus further stabilizing PCNA. These results suggested that simulated p-KAP1 could stabilize PCNA expression by competitively inhibiting CUL4A ubiquitin degradation.

### Simulated p-KAP1 (S824) improved long-term neurological deficits after cerebral I/R

**Fig. 6:**
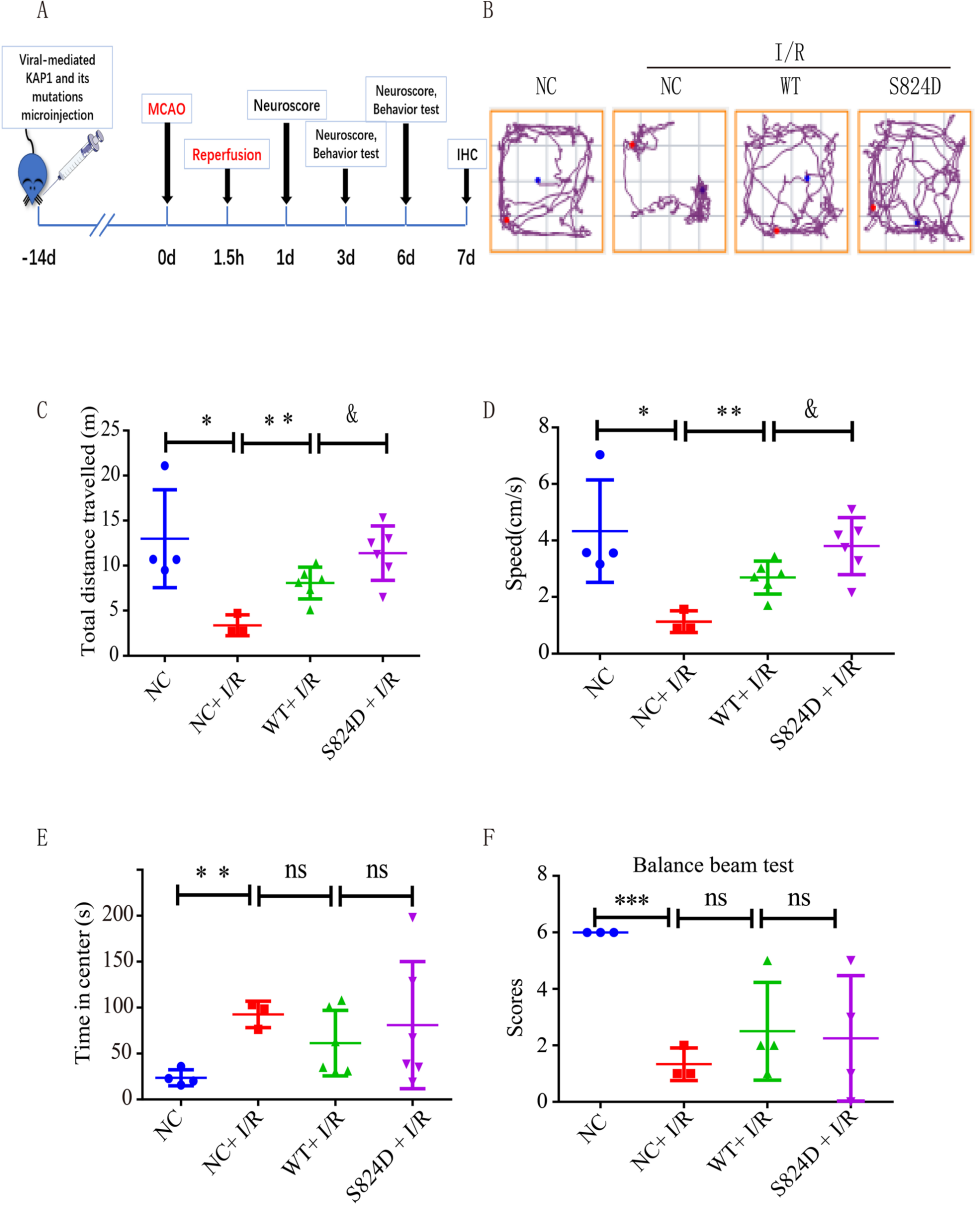
Simulated p-KAP1 (S824) improved locomotor activity of rats subjected to cerebral I/R. **A** Experimental design was illustrated schematically. A total of 50 rats were stochastic fall into 5 groups: the (1) NC (Scramble AAV 2/9), (2) NC + I/R (Scramble AAV 2/9 + I/R), (3) WT + I/R (AAV2/9-KAP1 + I/R), (4) S824D(AAV2/9-KAP1-S824D + I/R), (5) S824A(AAV2/9-KAP1-S824A + I/R) groups. Adeno-associated virus-mediated KAP1 and its mutation overexpression were administered 14 days before. After ischemia for 1.5 h, the rats were under reperfusion by pulling out the plug. **B**–**E** Effects of KAP1 and its mutations on locomotor activity of rats subjected to I/R. The total distance, speed, and time in the center were investigated by an open field test. Data were expressed as mean ± SEM (*n* = 3–6), ^*^P < 0.05, ^**^P < 0.01 versus NC + I/R group; ^&^*P* < 0.05 versus WT + I/R group. **F** A balance beam test was performed on the sixth day after cerebral ischemia. Data were expressed as mean ± SEM (n = 3–4), ^***^P < 0.001 versus NC + I/R group

We explored whether p-KAP1 (S824) could achieve a long-term therapeutic effect on rats against cerebral I/R-induced behavioral deficits. Behavioral tests were performed 6 days after cerebral I/R. A schematic of the study design is depicted in Fig. 6A. In the open-field test, the locomotor activity levels were severely impaired in the NC group after I/R. Still, treatment with KAP1-WT and its S824D mutation increased the recovery of neurological function as assessed by the total distance and speed (Fig. 6B–D). The KAP1-S824D mutant was better than KAP1-WT. But there was no difference in time in the center of the open-fieldtest and balance beam test between KAP1-S824D mutant and KAP1-WT (Fig. 6E and F). Collectively, these data indicated that mimicked p-KAP1 achieved a long-term protective effect against cerebral I/R injury.

## Discussion

**Fig. 7:**
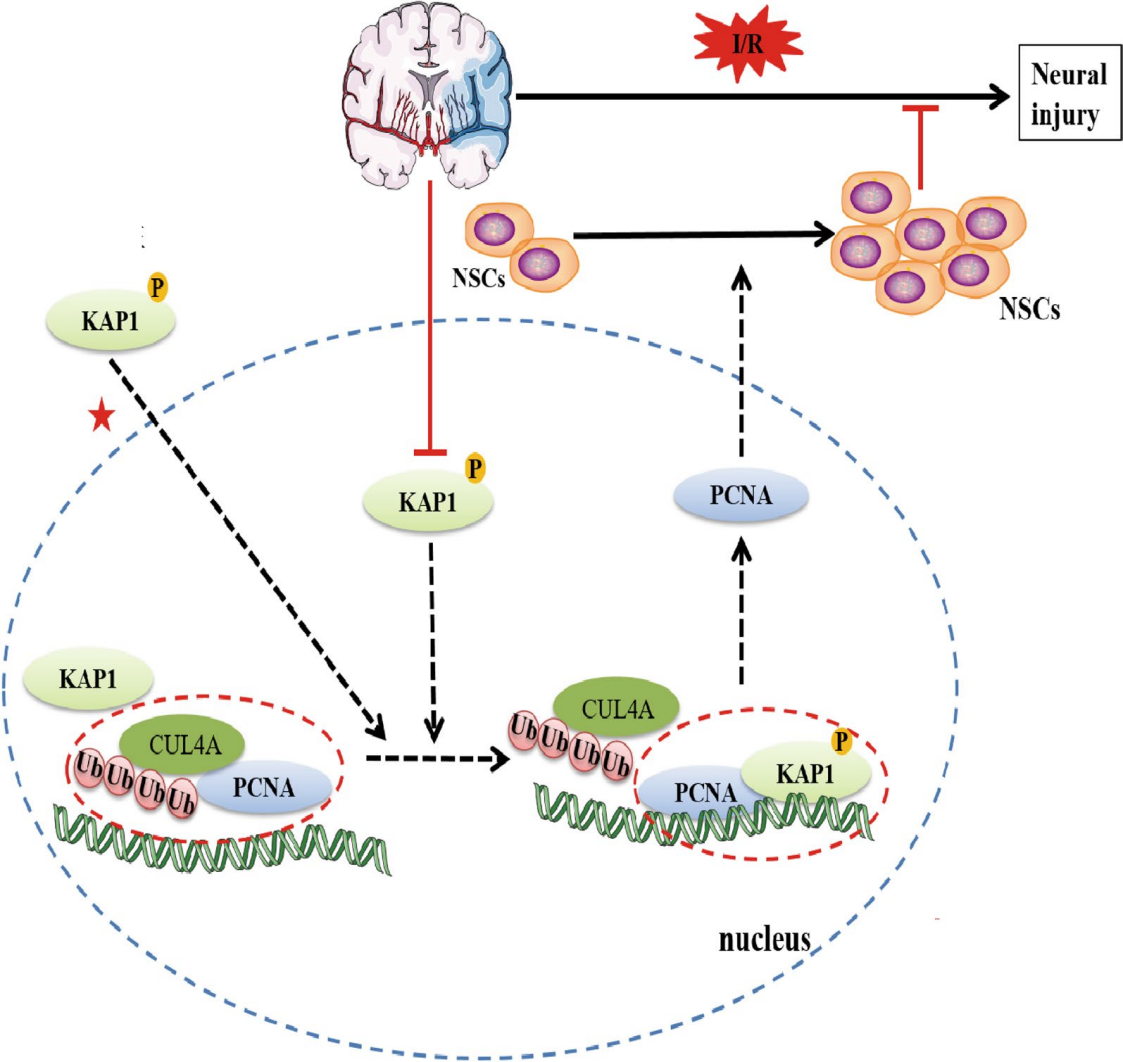
Schematic diagram of KAP1 phosphorylation during cerebral I/R involved in endogenous NSCs proliferation and apoptosis is summarized. Simulated p-KAP1 promoted the proliferation of endogenous neural stem cells (NSCs) and the expression of PCNA in cerebral I/R. Simulated p-KAP1 bound to PCNA, inhibiting CUL4A-mediated ubiquitination degradation to PCNA. This finding suggests that targeting the KAP-1/PCNA signaling pathway could enhance the neurogenesis of endogenous NSCs in the management of brain diseases

Simulated p-KAP1(S824) promoted the survival and proliferation of endogenous NSCs through increasing and stabilizing PCNA expression against cerebral I/R injury. We found no change in total KAP1 expression in endogenous NSCs in cerebral I/R, but its p-KAP1 level was decreased. The simulated p-KAP1 could promote the proliferation of NSCs, reduce their apoptosis and improve the behavioral deficits of rats after cerebral I/R. In the proposed mechanism, p-KAP1 increased the binding of PCNA and competitively inhibited the ubiquitination of PCNA by CUL4A in NSCs (Fig. 7). Overall, the results show that p-KAP1 is promising for use as a new strategy to promote NSCs proliferation by the KAP1/PCNA signaling pathway.

Enhancing the proliferation of endogenous NSCs is an essential component of stroke treatment. KAP1 is a transcription cofactor [33], and its structural domain, mainly localized in the cellular nucleus, can bind to members of the zinc finger protein family (ZFPs) [15, 34–36]. The function of KAP1 depends mainly on its phosphorylation state; p-KAP1 participates in human cytomegalovirus activation, and inhibition of p-KAP1 can depress viral replication [37]. Early infection with human adenoviruses (HAdV5) increases levels of p-KAP1 in cells and creates favorable conditions for viruses to replicate and enhance viral gene transcription [38]. Similarly, p-KAP1 is involved in Epstein Barr virus replication [39]. Phosphorylation of KAP1 at S473 and S824 is involved in the regulation of DNA damage repair [40]. In addition, p-KAP1 (S824) regulates the biological behavior of malignant tumors. Compared with noninvasive breast cancer, both KAP1 and p-KAP1 (S824) are increased in highly invasive breast cancer tissues [41]. Melanoma antigen encoding genes (melanoma-associated antigen encoding gene-C2, MAGE-C2) can bind KAP1, increasing p-KAP1 (S824) levels, promoting tumor cell proliferation and restoration of the genes that inhibit apoptosis, accelerating overall malignant tumor progression [42]. Although there have been many studies of p-KAP1 on viruses and cancers, little is known of KAP1 function in the neuronal system. KAP1 is highly expressed in CNS [43]. Herein, in a model of rat MCAO, we found that p-KAP1 (S824) decreased significantly despite the unchanged total expression of KAP1. Subsequently, using an in vitro model of OGD/R, we also found a significantly reduced level of p-KAP1 (S824) in C17.2 cells*.* We speculated whether simulated p-KAP1 could restore the deficit of neural function induced by cerebral I/R. Transient transfection of p-KAP1 (S824) mimics plasmid significantly reversed OGD/R-depressed cell proliferation. These results were further validated by the in vivo Ki-67 and Nestin colocalization assay. Thus, simulated p-KAP1 could enhance the proliferation of endogenous NSCs after cerebral I/R. Our previous research found that Momordica charantia polysaccharides promote endogenous NSC proliferation through the SIRT1/β-catenin pathway after cerebral I/R injury [44]. Theaflavin enhanced NSC proliferation by targeting miRNA-128-3p and further activating the Nrf2 pathway to reduce oxidative stress [45]. This study demonstrated that p-KAP1 promotes NSCs proliferation by modulating the PCNA pathway in cerebral I/R injury.

PCNA is a core component of the eukaryotic replication complex with a unique ring tertiary structure. PCNA can bind to different replication-related proteins to coordinate the DNA replication process [26, 46–48]. PCNA also acts as a functional conversion factor, interacts with various cytokines through different regulatory modes, and participates in many important cellular events, including DNA damage repairment and refinement, cell cycle regulation, and apoptosis [46, 49–51]. As an indicator of cell proliferation, PCNA correlates with the occurrence and development of diseases linked to cell proliferation [51–53]. Therefore, we inferred that the effects of p-KAP1 on NSC proliferation might be related to the regulation of PCNA. Western blotting revealed that the p-KAP1 mimic could improve OGD/R-induced suppression of PCNA, but it was unclear how this regulation occurs. Overall, there is significant interest in the potential relationship between p-KAP1 and the expression of PCNA in NSCs subjected to cerebral I/R. Suk Min Jang et al. reported binding of KAP1 to PCNA and p-KAP1(S473) enhanced this binding [26]. Here, we confirm the interaction of KAP1 and PCNA. The association of KAP1 with PCNA significantly decreased in NSCs after cerebral I/R, while p-KAP1(S824) enhanced this interaction. KAP1 can also act as an E3 ubiquitin ligase in some cellular pathways [54–57], so we asked if it was an E3 ubiquitin ligase of PCNA. Unexpectedly, the increased association between KAP1 and PCNA did not lead to a decrease in the expression of PCNA. Therefore, we deduced that regulation of PCNA may be indirect, where p-KAP1 influences other ubiquitin enzymes involved in mediating the stability of PCNA. Residue Tyr-322 of p-PCNA by the EGF receptor (EGFR) protected PCNA from poly-ubiquitylation and degradation mediated by E3 ubiquitin ligase CUL4 and promoted cell proliferation [58]. Molecular docking revealed that KAP1 could bind to PCNA at Arg-210, which is adjacent to Tyr-211. The interaction of KAP1 with PCNA may disrupt the interaction between CUL4A and PCNA, thereby influencing the proteolysis of PCNA mediated by CUL4A. To test this idea, we employed an immunoprecipitation experiment. As shown in Fig. 3F, the binding of CUL4A to PCNA increased in C17.2 cells exposed to OGD/R, while the use of the p-KAP1 mimic suppressed the interaction of CUL4A with PCNA. In the rat MCAO model, we also observed that virus-mediated KAP1 mutant (S824D) promoted the expression of PCNA; this was accompanied by a reduction in colocalization of PCNA and CUL4A. This result indicated that simulated p-KAP1 could competitively inhibit the interaction of PCNA with its E3 ubiquitin ligase CUL4A.

PCNA poly-ubiquitination has been shown to inhibit cell proliferation and regulate cell differentiation, apoptosis, and other processes [50, 59]. The decreased expression of PCNA induced by cerebral I/R could be reversed by the p-KAP1mimic, possibly due to the altered stability of PCNA. To test this, we detected the mRNA level of PCNA in C17.2 cells after OGD/R. No significant difference was found between the normoxia and OGD/R groups (Fig. 4A). The expression of PCNA decreased in C17.2 cells after OGD/R, and pre-treatment of MG132 (a proteasome inhibitor) reversed OGD/R-induction of the decrease of PCNA. Thus, we preliminarily concluded that OGD/R treatment caused the ubiquitination of PCNA. Mono-ubiquitination and poly-ubiquitination could happen at the K164 site of PCNA. Mono-ubiquitination of PCNA by E3-ubiquitin ligase Rad18 can promote translesion synthesis, and K63-linked poly-ubiquitination of PCNA by Rad6 can promote template switching [60]. Compared with the poly-ubiquitination formed via the K48 junction, mono-ubiquitination and poly-ubiquitination via K63 junctions generally do not cause protein degradation but instead alter the function of the substrate or mediate the interaction between the proteins [61–64]. We next measured the ubiquitination of PCNA in NSCs subjected to cerebral I/R. The results showed that ubiquitination of PCNA (K48 and K63) in C17.2 cells increased significantly, and the use of the p-KAP1 mimic could reverse this poly-ubiquitination (K48). This was consistent with what we have shown above, that simulated p-KAP1 promoted binding to PCNA, thereby competitively inhibiting the association of PCNA with CUL4A, involved in mediating the proteolysis of PCNA. The results show that PCNA ubiquitination related to p-KAP1 is mainly through the pathway of K48-polyubiquitination that mediates degradation by the proteasome system. However, the K63-polyubiquitination of PCNA also increased in C17.2 cells after OGD/R. PCNA plays a vital role in DNA damage repair, and its K63-polyubiquitination is generally increased in the DNA damage response by transducing the damage signal for downstream factors [65], so it may modulate some signal pathways. Given this, the effects of p-KAP1 on the apoptosis of C17.2 cells exposed to OGD/R were also detected. The results showed that p-KAP1 mimic might reverse apoptosis of OGD/R-induced C17.2 cells through the pathways involved in Bcl-2/Bax (Fig. 3C). C17.2 cells are immortalized mouse NSCs (mNSCs). Their stemness genes permit the cells to be efficiently grown in homogenous and viable quantities to ensure reproducible integration patterns [66]. The clone C17.2 has been affirmed (after many years of investigation) to fulfill the in vitro and in vivo operational definitions of a stem cell, including the ability not only to give rise to multiple neural cell types throughout the neural axis during development but also to reconstitute those regions when they have been perturbed [67–69]. This NSC clone also expresses all commonly accepted NSC markers (e.g., nestin, musashi, vimentin, A2B5) and can, under serum-free mitogensupplemented culture conditions, form neurospheres. In this study, we performed plasmid transfection on C17.2 cells rather than primary NSCs, as primary NSCs were unstable and difficult to be transfected. Compared with primary NSCs, C17.2 cells could remain stable through multiple passages without losing multipotency, self-renewal capacity, engraftability, or the ability to differentiate appropriately in response to regional cues throughout the neuraxis, emulate the biology of endogenous NSCs. Mechanistically, the results in C17.2 cells can verify our hypothesis, but primary NSCs are better for mimicking the proliferation of NSCs.

An improvement in neurological deficits will result in a better prognosis. We next detected the long-term effects of simulated p-KAP1 (S824) after cerebral I/R. We used a strategy of AAV2/9-mediated p-KAP1 14 days before the model and verified the success of lateral ventricular injection. After transfection of rats with S824A mutant viral vector, the S824A mutant rats die due to intestinal obstruction or vascular atrophy. We found that the rats treated with the S824D virus vector showed good functional recovery after cerebral I/R. Regarding behavioral indicators after cerebral I/R, rats in the S824D group showed higher locomotor activity. Still, there was no effect on time in the center and no effect on the results of the balance beam test. These data suggested the improvement in long-term neurological deficits by simulating p-KAP1.

## Conclusion

In summary, we revealed the unique role of KAP1 in regulating the neurogenesis of endogenous NSCs after cerebral I/R through the KAP1/PCNA signaling pathway. Simulated p-KAP1 could reverse cerebral I/R-induced neural deficit and enhance the  proliferation of endogenous NSCs by improving the stable expression of PCNA and reducing the apoptosis rate. Strategies targeting p-KAP1 should be helpful for the prevention or treatment of ischemic stroke. Future studies are required to determine the details of the specific regulatory mechanism of the apoptosis of NSCs by p-KAP1.

## Supplementary Information

**Additional file 1**. Supplementary figures.

## Data Availability

The datasets used and/or analyzed during the current study are available from the corresponding author on reasonable request.

## Abbreviations

KAP1: KRAB domain protein 1
p-KAP1: Phosphorylation of KAP1
S824: Serine 824 site
PCNA: Proliferation cell nuclear antigen
NSCs: Neural stem cells
I/R: Ischemic/reperfusion
KRAB: Krueppel-associated box
ZFPs: Zinc finger proteins
TRIM28: Tripartite motif-containing protein 28
TIF1β: Transcriptional intermediary factor 1β
OGD/R: Oxygen glucose deprivation/reperfusion
GFP: Green fluorescent protein
MCAO: Middle cerebral artery occlusion
CCA: Common carotid artery
ECA: External carotid artery
ICA: Internal carotid artery
CCK-8: Cell counting kit-8
SVZ: Subependymal ventricular zone
