# Supplementary material for: KAP1 phosphorylation promotes the survival of neural stem cells after ischemia/reperfusion by maintaining the stability of PCNA
Source: Stem Cell Res Ther. 2022 Jul 7;13:290. doi: 10.1186/s13287-022-02962-5 (PMC9264526; doi:10.1186/s13287-022-02962-5)
Supplement: Supplementary file 1 — Additional file 1. Supplementary figures. [file 13287_2022_2962_MOESM1_ESM.docx]

**Supplement Material**

1. **Statistics of immunofluorescence intensity of KAP1 and p-KAP1.**


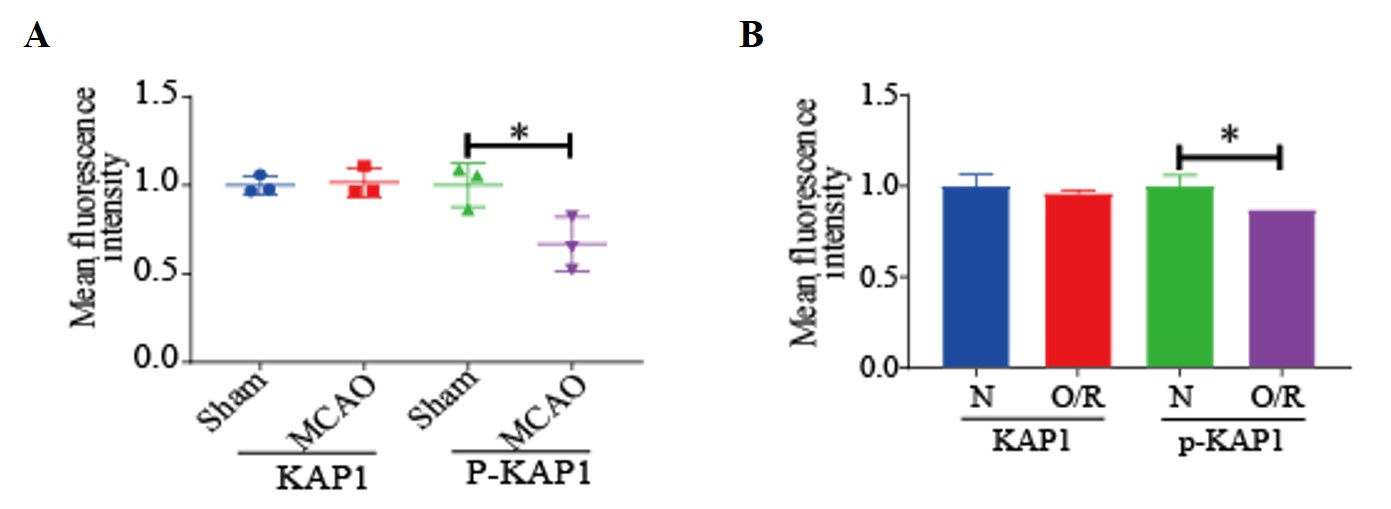


**Fig. S1 Correlation Fluorescence Intensity Statistics.** (A) The phosphorylation and protein expression of KAP1 in SVZ of rat subjected to I/R were analyzed by immunofluorescence staining (Figure 1B). Data were quantified as mean ± SEM (n = 3), ^*^*P* < 0.05 versus sham. (B) Immunofluorescence staining Data were quantified as mean ± SEM (n = 3) to detect the phosphorylation and protein expression (Figure 1E), ^*^*P* < 0.05 versus normoxia group.

1. **Western blot of cell viability-related proteins of C17.2 cells after OGD/R.**


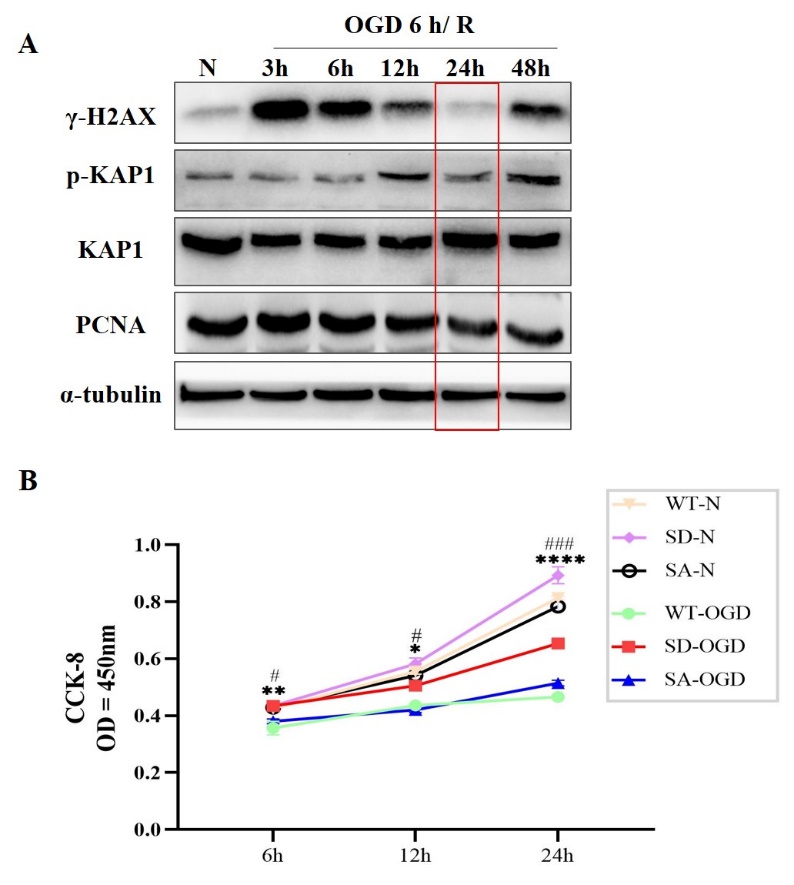


**Fig. S2 Cell viability-related indicators of C17.2 cells after OGD/R.** (A) Expression of related proteins of C17.2 subjected to OGD/R at the reperfusion different times.

1. **Statistics of BrdU immunofluorescence intensity.**


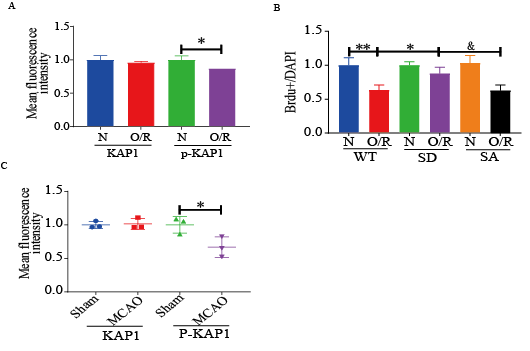


**Fig. S3 Correlation Fluorescence Intensity Statistics.** Brdu immunofluorescence staining was performed to evaluate the C17.2-NSCs proliferation. Data were quantified as mean ± SEM (n = 3), ^*^*P* < 0.05, ^**^*P* < 0.01 versus WT and O/R group (Figure 2A)；^&^ *P* < 0.05 versus SD and O/R group, 200 ×, scale bar = 50 μm.

1. **Proliferation curve of C17.2 cells tested by CCK-8 assay at the differences over time of the various groups.**


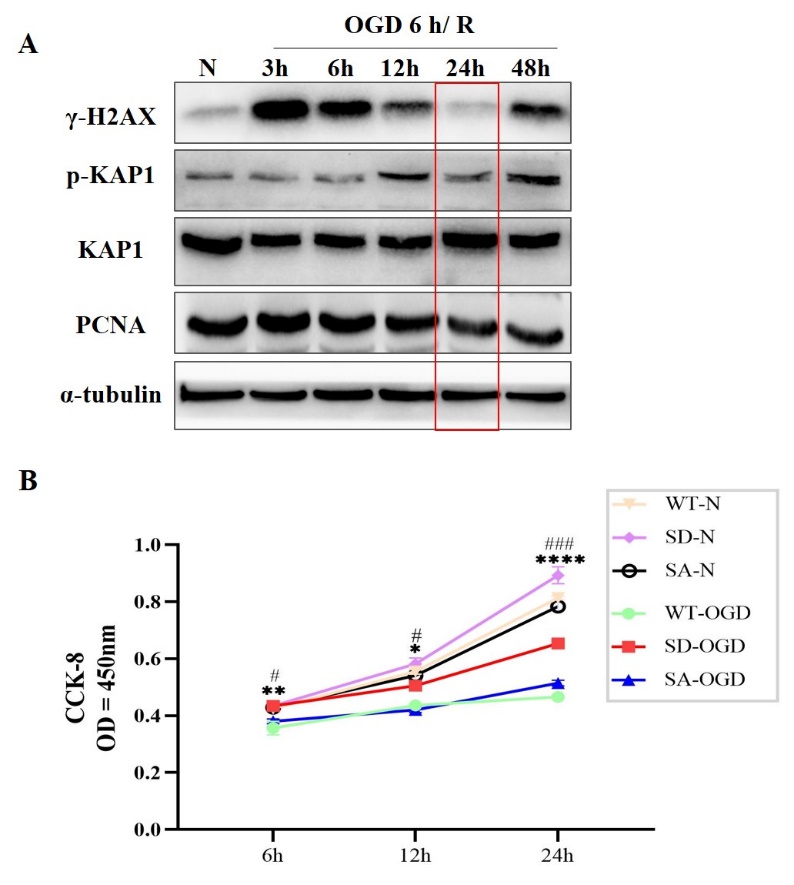


**Fig. S4 Cell viability of C17.2 cells after OGD/R.** A proliferation curve of C17.2 cells to evaluate proliferation by CCK-8 assay at the differences over time of the various groups. Groups: Normal groups (WT-N, SD-N, SA-N); The OGD/R groups (WT-OGD, SD-OGD, SA-OGD). Data were presented as mean ± SEM (n = 3), ^*^*P* < 0.05, ^**^*P* < 0.01, ^****^*P* < 0.0001, SD-OGD versus WT-OGD; ^#^*P* < 0.05, ^###^*P* < 0.001, SD-OGD versus SA-OGD.
